# Addressing Chronic Gynecological Diseases in the SARS-CoV-2 Pandemic

**DOI:** 10.3390/medicina59040802

**Published:** 2023-04-20

**Authors:** Maria-Loredana Țieranu, Nicoleta Alice Dragoescu, George-Lucian Zorilă, Anca-Maria Istrate-Ofițeru, Cătălina Rămescu, Elena-Iuliana-Anamaria Berbecaru, Roxana Cristina Drăguşin, Rodica Daniela Nagy, Răzvan Grigoraș Căpitănescu, Dominic-Gabriel Iliescu

**Affiliations:** 1 Doctoral School, University of Medicine and Pharmacy of Craiova, 20039 Craiova, Romania; 2 Department of Obstetrics and Gynecology, Emergency County Hospital of Craiova, 200642 Craiova, Romania; 3 Department of Anesthesiology and Intensive Care, Emergency County Hospital of Craiova, University of Medicine and Pharmacy of Craiova, 200349 Craiova, Romania; alice.dragoescu@yahoo.com; 4 Department of Obstetrics and Gynecology, University of Medicine and Pharmacy of Craiova, 200349 Craiova, Romania; 5 Department of Histology, University of Medicine and Pharmacy of Craiova, 200349 Craiova, Romania; 6 Research Centre for Microscopic Morphology and Immunology, University of Medicine and Pharmacy of Craiova, 200349 Craiova, Romania

**Keywords:** SARS-CoV-2 pandemic, gynecologic surgery, vaccination, statistical studies

## Abstract

*Introduction*: the COVID-19 pandemic has had a considerable impact on healthcare systems worldwide. Since the actual influence of the pandemic on gynecological care is still unclear, we aim to evaluate the effect of the SARS-CoV-2 pandemic on gynecological procedures compared to the pre-pandemic period in Romania. *Materials and Methods*: this is a single-center retrospective observational study, involving patients hospitalized in the year before the SARS-CoV-2 pandemic (PP), in the first year of the pandemic (P1), and in the second year of the pandemic until February 2022 (P2). The percentages of interventions were analyzed globally but also according to the type of surgery applied on the female genital organs. *Results*: during pandemic, the number of gynecological surgeries dropped considerably, by more than 50% in some cases, or even decreased by up to 100%, having a major impact on women’s health, especially in the first year of the pandemic (P1), before slightly increasing in the post-vaccination period (PV). Surgically treated cancer cases dropped by over 80% during the pandemic, and the consequences of this will be seen in the future. *Conclusions*: the COVID-19 pandemic played an important part in gynecological care management in the Romanian public health care system, and the effect will have to be investigated in the future.

## 1. Introduction

The coronavirus pandemic, or the COVID-19 pandemic, is a global health problem caused by severe acute respiratory syndrome coronavirus 2 (SARS-CoV-2). This virus was initially identified from an outbreak in Wuhan, China, in December 2019, with the ability to spread rapidly in Asia and later worldwide. The World Health Organization (WHO) declared the outbreak on 30 January 2020, a public health emergency of international concern, and on 11 March 2020, it was stated a pandemic [1,2]. A large number of deaths were caused worldwide by the pandemic. By 11 November 2022, this virus had infected over 634 million patients, and 6.6 million were confirmed dead, making this virus one of the deadliest in history [3]. The public health response to the pandemic was to minimize the viral spread by imposing restrictions, preventing the overloading of the healthcare system, and by purchasing medical supplies for protection [3].

Most of the registered cases were associated with mild or even absent symptoms, but the infection, in some cases, led to severe respiratory diseases and even multiorgan failure, especially in people with comorbidities [3]. The researchers responded to this pathology with a high level of transparency. They exchanged information and data to better understand the physiopathology, epidemiology, and clinical implications of this problem, with the common goal of developing vaccines and mitigation strategies, and designing appropriate therapies [3]. Still, there is concern that current medical practice suffers from fewer resources, restrictions and population fear with respect to the provision of health care services.

Multiple studies with different approaches have been carried out to understand the impact of COVID-19 on the maternal–fetal binomial.

Karimi L. et al. reported that the mortality rate caused by SARS-CoV-2 infection in pregnant and postpartum women was 1.3%, and the rate of severe pneumonia was reported to be between 1% and 14%; therefore, maternal death was in agreement with studies reported for other severe lower respiratory tract viral infections [4,5,6,7,8,9,10,11], but other studies supported the mortality rate of pregnant women infected with SARS-CoV-2 being similar to non-pregnant women of reproductive age [12,13,14]. This was explained by the younger age of the pregnant women without associated comorbidities, compared to older patients in whom related comorbidities were present [15]. Additionally, other studies have suggested that immunological changes during pregnancy may be the cause of maternal vulnerability to the virus and may significantly affect the immune response against SARS-CoV-2 [16]. Comorbidities in pregnant women, e.g., presence of pneumonia, cardiopulmonary complications, multiorgan failure, especially in the second or third trimester of pregnancy, have frequently led to maternal death [17]. La Verde M et al. showed that in the majority of pregnant women infected with SARS-CoV-2 who died, this occurred postpartum, especially in those who were associated with diabetes or obesity, but the evidence reporting the characteristics of maternal mortality and morbidity with SARS-CoV-2 is of limited and low quality at this time [18].

However, few studies have been dedicated to investigating the pandemic’s effect on gynecological care, which may have overwhelming implications for the health of the female population as well as physical and psychological trauma in the general population [3,19,20,21,22].

Our study aimed to assess the influence of the SARS-CoV-2 pandemic on the profile and relative number of gynecological procedures during the COVID pandemic period and to report the resumption rates after the introduction of the vaccine. A secondary objective was to assess the condition of patients during the study periods by examining the biological state at hospitalization.

## 2. Materials and Methods

This was a single regional center observational retrospective study conducted in Romania from March 2019 to February 2022 in the Obstetrics and Gynecology department of the Clinical Emergency County Hospital Craiova, which included 1978 eligible patients hospitalized and treated for gynecological conditions.

All these patients were distributed according to the time period in which they were hospitalized. We considered 3 periods for analysis: the first period was between March 2019 and February 2020—the pre-pandemic year, before the SARS-CoV-2 pandemic (PP); the second period, between March 2020 and February 2021, represents the first year of the SARS-CoV-2 pandemic (P1); the third analyzed period, between March 2021 and February 2022, corresponds to the second year of the SARS-CoV-2 pandemic (P2). The end of the P1 period (starting with December 2020) and the entire P2 period took place simultaneously with the post-vaccination period (PV).

All cases were divided according to the period (PP, P1, P2) and month of study for comparative studies, and we were able to investigate the distribution of gynecological surgical interventions. All numerical values, percentages, and graphics were acquired and processed using Microsoft Excel 2010.

## 3. Results

Gynecological surgical procedures were represented by interventions for ovarian pathology—unilateral adnexectomy (UA), bilateral adnexectomy (BA), pregnancy curettage or incomplete abortion (PC); biopsies—hemostatic curettage or endometrial biopsy (EB); biopsies performed for various vaginal or vulvar lesions—vaginal biopsy (VB), vulvar biopsy (VuB), polypectomies (P); interventions performed for various uterine pathologies (leiomyoma/adenomyosis/cancer/ovarian pathologies in perimenopause)—total hysterectomy with unilateral adnexectomy (TH-UA), total hysterectomy with bilateral adnexectomy (TH-BA), subtotal hysterectomy (SH), vaginal hysterectomy (VH), radical hysterectomy (RH), myomectomies (M); laparoscopic interventions—exploratory laparoscopy (EL), laparoscopic myomectomy (LM), laparoscopic salpingectomy (LS), unilateral laparoscopic cystectomy (ULC), bilateral laparoscopic cystectomy (BCL), exploratory laparotomies (ExL); and interventions performed for tubal pathology (tubal ectopic pregnancy or pelvic inflammatory disease)—tubal ectopic pregnancy salpingectomy (TEPS), bilateral salpingectomy (BS) and unilateral salpingectomy (US). All patients included in the study gave their written and verbal consent to participate in this study.

**Figure 1:**
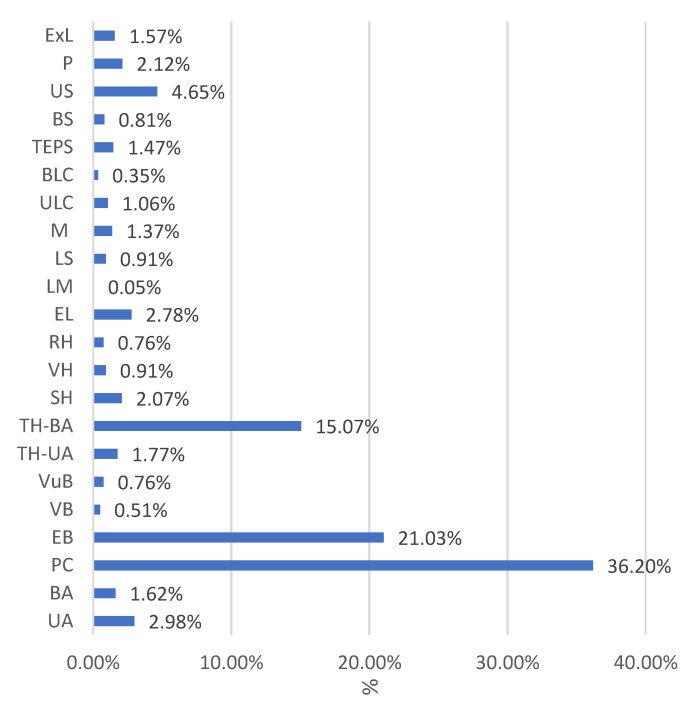
The rates of each intervention type. UA: unilateral adnexectomy; BA: bi-lateral adnexectomy; PC: pregnancy curettage; EB: endometrial biopsy; VB: vaginal biopsy; VuB: vulvar biopsy; TH-UA: total hysterectomy with unilateral adnexectomy; TH-BA: total hysterectomy with bilateral adnexectomy; SH: subtotal hysterectomy; VH: vaginal hysterectomy, RH: radical hysterectomy; EL: exploratory laparoscopy; LM: laparoscopic myomectomy; LS: laparoscopic salpingectomy; M: myomectomies; TEPS: tubal ectopic pregnancy salpingectomy; BS: bilateral salpingectomy; US: unilateral salpingectomy; P: polypectomy; ExL: exploratory laparotomy.

The rates of each intervention type during the studied period were: interventions for ovarian pathology: UA—2.98%, BA—1.62%; PC—36.20%; EB—21.03%; biopsies performed for various vaginal or vulvar lesions: VB—0.51%, VuB—0.76%, P—2.12%; interventions performed for various uterine pathologies (leiomyoma/adenomyosis/cancer/ovarian pathologies in perimenopause): TH-UA—1.77%, TH-BA—15.07%, SH—2.07%, VH—0.91%, RH—0.76%, M—1.37%; laparoscopic interventions: EL—2.78%, LM—0.05%, LS—0.91%, ULC—1.06%, BCL—0.35%; interventions performed for tubal pathology (ectopic tubal pregnancy or pelvic inflammatory disease): TEPS—1.47%; BS—0.81%, US—4.65%, ExL—1.57% (Figure 1).

### 3.1. Interventions for Ovarian Pathology

**Figure 2:**
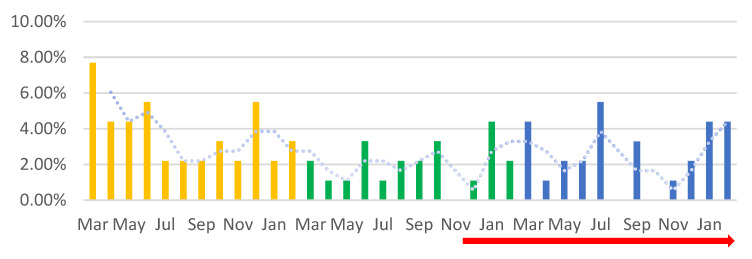
The percentage of adnexectomies by month among the total number of adnexectomies (%). A downward trendline can be observed for the percentage of adnexectomies performed during the SARS-CoV-2 pandemic. Orange—PP: the year before the SARS-CoV-2 pandemic; green—P1: the first year of the SARS-CoV-2 pandemic; blue—P2: the second year of the SARS-CoV-2 pandemic; red arrow—PV: post-vaccination period.

**Figure 3:**
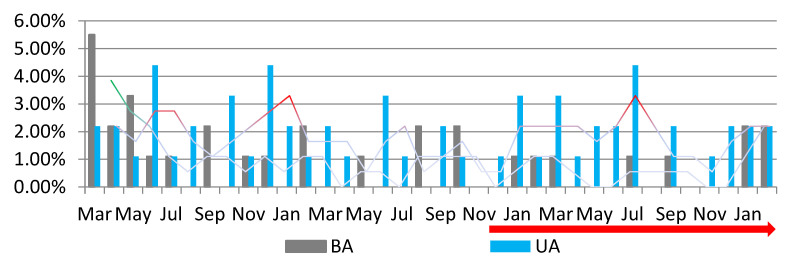
The percentage value of BA (the gray columns) and UA (the blue columns) among the total number of adnexectomies performed (%). A downward trendline can be observed regarding the percentage of adnexectomies performed during the SARS-CoV-2 pandemic. The green peak coincides with the period with the highest number of BAs, and the red peaks coincide with the periods with the most UA. BA: bilateral adnexectomy; UA: unilateral adnexectomy; red arrow—PV: post-vaccination period.

A total of 91 adnexectomies were performed, representing 4.60% of the total surgical interventions performed in the three periods included in the study. Out of these, 1.62% were bilateral adnexectomies (BA), and 2.98% were unilateral adnexectomies (AU). The highest number of adnexectomies was performed in PP—45; fewer adnexectomies were performed in P1—24.13%—and P2—30.86%. In PP, 43.96% were BA and 56.03% were UA (orange columns); in P1, 31.53% were BA and 68.46% were UA; the number of adnexectomies performed increased in P2 compared to P1 (30.86%), with 24.64% being BA and 74.64% being UA. Thus, it can be observed that the lowest number of adnexectomies was performed in P1 (Figure 2). We observed that most of the BAs performed occurred in PP, before a drastic decrease in both BA and UA procedures in P1, with an upward trend in P2 (PV), with the highest number of UA being recorded in July P2 (PV) (red peaks) (Figure 3). In the current study, a decrease in the total number of adnexectomies, by 20.87% in P1 and by 14.14% in P2 compared to PP, can be observed. Analyzing each type of adnexectomy, we observed that the number of BA decreased by more than half in P1 and P2 compared to PP, and the number of UA was reduced by half in P1 and by a quarter in P2 compared to PP.

### 3.2. Pregnancy Curettage or Incomplete Abortion

**Figure 4:**
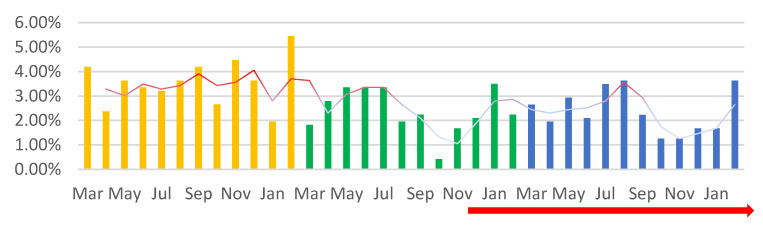
The percentage value of the pregnancy curettage among the total number of surgical interventions (%). A downward trendline can be observed for the percentage of pregnancy curettage procedures performed during the SARS-CoV-2 pandemic. Orange—PP: the year before the SARS-CoV-2 pandemic; green—P1: the first year of the SARS-CoV-2 pandemic; blue—P2: second year of the SARS-CoV-2 pandemic; red arrow—PV: post-vaccination period.

A total of 716 pregnancy curettage procedures were performed, representing 36.20% of the total surgeries performed in the three periods included in this study. Of these, 42.74% were performed in PP, 28.77% were performed in P1, and 28.49% were performed in P2, with a sharp decline in the pandemic. Thus, we observe that the number of pregnancy curettages reduced by 32.69% in P1 and 33.35% in P2 compared to PP The pregnancy curettages decreased by approximately one-third compared to PP in each year of the pandemic. The most affected months of the pandemic were October 2020 (2.65%), followed by October and November 2021 (1.26%), with an increase in the number of PCs in PV (red peaks), especially in August of P2 (3.63%) (Figure 4).

### 3.3. Hemostatic Curettage or Endometrial Biopsy, Biopsies Performed for Various Vaginal or Vulvar Lesions and Polypectomies

**Figure 5:**
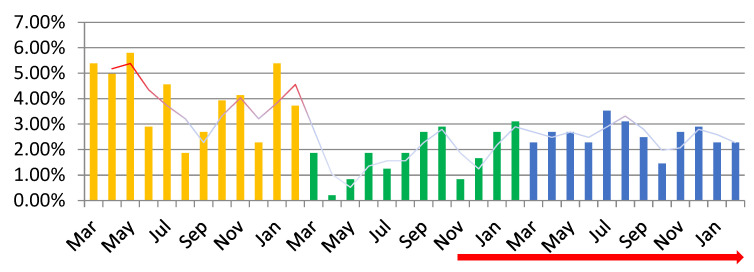
The total percentages of biopsies/polypectomies among the total number of procedures of this class (%). It can be observed that the fewest biopsies/polypectomies were performed in P1, with a significant decrease in the percentage compared to PP. Orange—PP: the year before the SARS-CoV-2 pandemic; green—P1: the first year of the SARS-CoV-2 pandemic; blue—P2: the second year of the SARS-CoV-2 pandemic; red arrow—PV: post-vaccination period.

In the three study periods, 483 biopsies and polypectomies were performed, representing 24.42% of the total number of surgical interventions. These were assigned as follows: 86.13% were hemostatic endometrial curettage/endometrial biopsies (EB), 2.07% were vaginal biopsies (VB), 3.11% were vulvar biopsies (VuB), and 8.70% were polypectomies. Their distribution shows that 47.62% were performed in PP, 21.74% in P1, and 30.64% of biopsies and polypectomies were performed in P2. We noted a downward tendency in the rate of biopsies performed during P1, especially in April and November (Figure 5). The overall percentage of biopsies and polypectomies decreased by 54.35% in P1 and 35.66% in P2 compared to PP. When considering only the number of polypectomies, a decrease by 61.54% can be observed in P1 and 76.92% in P2 compared to PP. In the case of EB, decreases of 54.93% in P1 and 29.53% in P2 compared to PP can be observed. The cases of VB decreased by 50% in P2 compared to PP and P1, and the cases of VuB decreased by 42.76% in P1 and P2 compared to PP.

### 3.4. Interventions Performed for Various Uterine Pathologies (Leiomyoma/Adenomyosis/Cancer/Ovarian Pathologies in Perimenopause)

**Figure 6:**
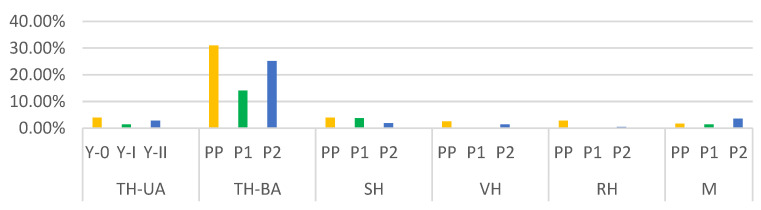
Distribution of hysterectomies by year. A significant percentage decrease in interventions for all categories of hysterectomies/myomectomies can be observed during the SARS-CoV-2 pandemic. Orange—PP: the year before the SARS-CoV-2 pandemic; green—P1: the first year of the SARS-CoV-2 pandemic; blue—P2: the second year of the SARS-CoV-2 pandemic; TH-UA: total hysterectomies with unilateral adnexectomies; TH-BA: total hysterectomies with bilateral adnexectomies; SH: subtotal hysterectomies; VH: vaginal hysterectomies; RH: radical hysterectomies; M: myomectomies.

During the three periods included in the study, 399 hysterectomies and 27 myomectomies were performed, representing 21.54% of the total number of interventions. Of these, 8.22% were total hysterectomies with unilateral adnexectomies (TH-UA), distributed as follows: PP, 3.99%; P1, 1.41%; and P2, 2.82%. Total hysterectomies with bilateral adnexectomies (TH-BA) were performed in 70.24% of cases, assigned as follows: PP, 30.99%; P1, 14.08%; P2, 25.17%. Subtotal hysterectomies (SH) represented 9.62% of the total number, distributed as follows: PP, 3.99%; P1, 3.76%; P2, 1.88%. Vaginal hysterectomies (VH) were performed in 4.23% of cases, distributed as follows: PP, 2.58%; P1, 0.23%; P2, 1.41%. Radical hysterectomies (RH) represented 3.52% of the cases, categorized as follows: PP, 2.82%; P1, 0.23%; P2, 0.47%. Myomectomies represented 6.62% of the interventions performed for various uterine pathologies, assigned to categories as follows: PP, 1.69%; P1, 1.41%; P2, 3.52% (Figure 6). For the cases of TH-UA, the number of interventions decreased by 64.67% in P1 and 29.33% in P2 compared to PP. In the case of TH-BA, the decrease was by 54.57% in P1 and 18.79% compared to PP. SH decreased by 5.77% in P1 and 52.89% in P2 compared to PP. The VH decreased by 91.09% in P1 and 45.35% in P2 compared to PP, and RH decreased by 91.85% in P1 and 83.34% in P2 compared to PP. M decreased by 16.57% in P1 and increased by 51.99% in P2 compared to PP.

### 3.5. Laparoscopic Interventions

Regarding laparoscopic interventions such as exploratory laparoscopies (EL), laparoscopic myomectomies (LM), laparoscopic salpingostomies (LS), bilateral laparoscopic cystectomy (BCL), and unilateral laparoscopic cystectomy (ULC), 102 interventions were performed, representing 5.16% of the total number of surgeries: 2.78% in PP, 0.76% in P1 and 1.62% in P2.

**Figure 7:**
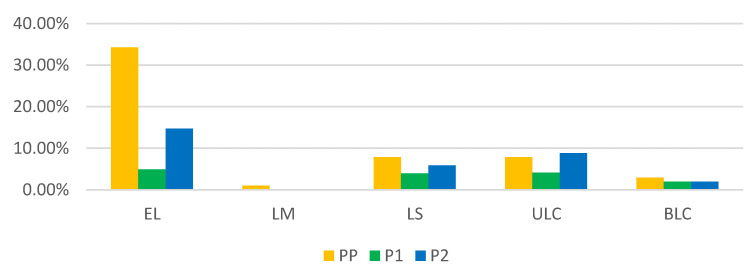
Distribution of laparoscopic interventions by year. There is a significant decrease in the number of surgeries in P1 (green columns) and P2 (blue columns). PP: the year before the SARS-CoV-2 pandemic; P1: the first year of the SARS-CoV-2 pandemic; P2: the second year of the SARS-CoV-2 pandemic; BCL: bilateral laparoscopic cystectomy; ULC: unilateral laparoscopic cystectomy.; LS: laparoscopic salpingostomy; LM: laparoscopic myomectomy; EL: exploratory laparoscopy.

BLC accounted for 6.86% of the total number of laparoscopic interventions performed, distributed as follows: 2.94% in PP, 1.96% in P1, and 1.96% in P2. ULC accounted for 20.79% of the laparoscopic interventions performed as follows: 7.84% in PP, 4.12% in P1, and 8.82% in P2. LS accounted for 17.65% of laparoscopic interventions, with 7.84% being performed in PP, 3.92% in P1, and 5.88% in P2. LM accounted for 0.98% of the total number of laparoscopic interventions performed in PP. EL accounted for 53.92% of the total number of laparoscopic interventions, with 34.31% being performed in PP, 4.90% in P1, and 14.71% in P2 (Figure 7). Regarding the number of laparoscopic interventions, a decrease in EL by 85.72% was observed in P1, and 57.13% in P2, compared to PP. All LM performed were in PP; therefore, there were 100% decreases in P1 and P2. A 50% decrease was observed in LS in P1 and a 25% decrease in P2 compared to PP. ULC decreased by 47.45% in P1 and increased by 11.12% in P2 compared to PP. BLC decreased by 33.33% in P1 and P2 compared to PP.

### 3.6. Exploratory Laparotomies

**Figure 8:**
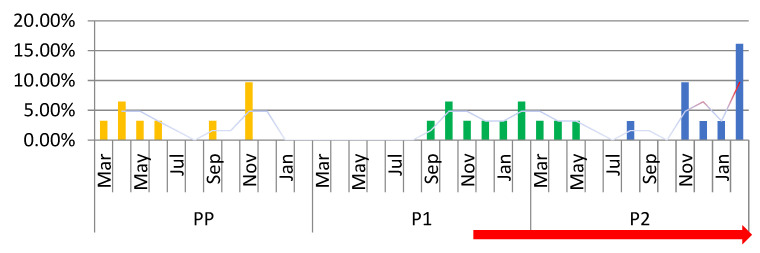
The total number of exploratory laparotomies by month. A significant decrease can be observed in the number of interventions in P1, especially at the beginning of the pandemic period. PP (orange columns): the year before the SARS-CoV-2 pandemic; P1 (green columns): the first year of the SARS-CoV-2 pandemic; P2 (blue columns): the second year of the SARS-CoV-2 pandemic. The red peaks represent the highest percentage of interventions performed; the red arrow shows the beginning of PV (the post-vaccination period).

In this study, 31 exploratory laparotomies (ExP) were performed, representing 1.57% of the total number of surgeries performed. In PP, 29.03% of the total number were performed, in P1, 25.81%, and in P2, 45.16% There was an extreme decline in the percentage of ExP, especially in P1. We observed a decrease to 0% at the beginning of P1, with a slight increase in the number of interventions towards the end of P1 and an increase at the end of P2, in PV (Figure 8). In this study, an 11.09% decrease can be observed in the number of laparotomies in P1 and a 35.72% increase in P2 compared to PP.

### 3.7. Interventions Performed for Tubal Pathology (Tubal Ectopic Pregnancy or Pelvic Inflammatory Disease)

**Figure 9:**
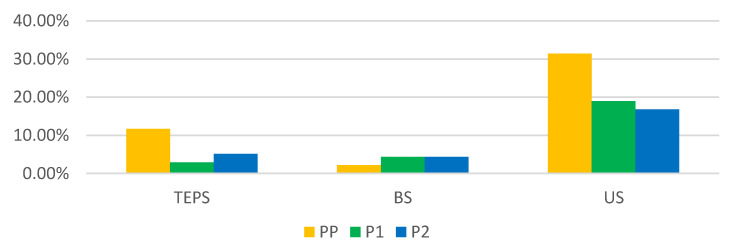
Distribution of salpingectomies by year. PP: the year before the SARS-CoV2 pandemic; P1: the first year of the SARS-CoV-2 pandemic; P2: the second year of the SARS-CoV-2 pandemic; US: unilateral salpingectomy; BS: bilateral salpingectomy; TEPS: tubal ectopic pregnancy salpingectomy.

During the study periods, 137 salpingectomies were performed, representing 6.93% of the total number of interventions. With respect to the period, 45.26% of tubal interventions were performed in PP out of the total number of interventions, distributed as follows: 11.68% of the total number of interventions were tubal ectopic pregnancy salpingectomies (TEPS), 2.19% were bilateral salpingectomies (BS) and 31.39% were unilateral salpingectomies (US); in P1, 26.28% of tubal interventions were performed from among the total number of interventions, distributed as follows: 2.92% TEPS, 4.38% BS and 18.98% US; and in P2, 26.28% of tubal interventions were performed, distributed as follows: 5.11% TEPS, 4.38% BS and 16.79% US (Figure 9). The number of TEPs performed decreased by 75% in P1 and 56.25% in P2 compared to PP. BS increased by 50% in P1 and P2 compared to PP, and US decreased by 39.53% in P1 and 46.51% in P2 compared with PP.

### 3.8. The Age of the Patients

**Figure 10:**
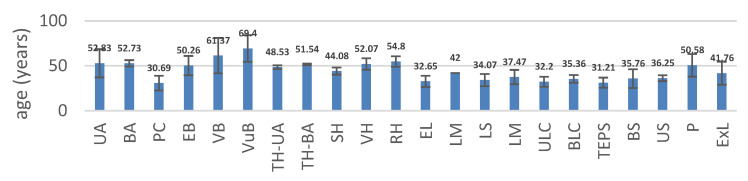
Mean age (years) of patients. UA: unilateral adnexectomy; BA: bilateral adnexectomy; PC: pregnancy curettage; EB: endometrial biopsy; VB: vaginal biopsy; VuB: vulvar biopsy; TH-UA: total hysterectomy with unilateral adnexectomy; TH-BA: total hysterectomy with bilateral adnexectomy; SH: subtotal hysterectomy; VH: vaginal hysterectomy, RH: radical hysterectomy; EL: exploratory laparoscopy; LM: laparoscopic myomectomy; LS: laparoscopic salpingostomy; M: myomectomies; TEPS: tubal ectopic pregnancy salpingectomy; BS: bilateral salpingectomy; US: unilateral salpingectomy; P: polypectomy; Elap: exploratory laparotomy.

The mean age of the patients included in the study ranged from 30.69 years (±8.22 years) for PC to 69.4 years (±14.91 years) for VuB-type interventions (Figure 10).

The average age of patients in whom BA/AU was performed was 52.83 years (±15.74 years). In PP, the average age was 55.85 years (±11.53 years), in P1, it was 53.6 years (±16.77 years old), and in P2, the average age of the patients was 48.75 years (±17.9 years).

Regarding the age of the patients in whom pregnancy curettage was performed, an average value of 30.69 years (±8.22 years) was noted. In PP, the average age was 31.93 years (±8.55 years), in P1, 29.84 years (±7.73 years old), and in P2, the average age of patients was 29.38 years (±7.86 years). Therefore, we note that the age of patients undergoing pregnancy curettage gradually decreased during the SARS-CoV-2 pandemic.

Regarding the average age of the biopsied patients, we noticed that the average age of all those who underwent endometrial biopsy was 50.26 years (±10.77 years). In PP, the average age was 50.59 years (±11.18 years); in P1, the average age was 51.21 years (±11.09 years), and in P2, the average age was 49 years (±9.77 years); the highest mean age of endometrial biopsied patients was in P1. The mean age of all patients undergoing vaginal biopsy was 61.37 years (±19.76 years); in PP, the mean age was 69.33 years (±28.29 years); in P1, it was 54.66 years (±13.79 years), and in P2 the average age was 59.50 years. The highest mean age of vaginal biopsy patients was recorded in PP. In the case of patients who underwent vulvar biopsy, their mean age was 69.40 years (±14.91 years); in PP, the mean age was 75.71 years (±10.62 years); in P1, it was 75.71 years (±10.62 years), and in P2, the average age was 68.25 years (±12.17 years). We thus observed that the highest average value of patient age was registered in PP. In P1 and P2, the ages of the patients were lower.

The mean age of hysterectomy patients varied as follows: TH-AU—PP: 50.31 years (±8.29 years), P1: 46.16 years (±13.22 years), P2: 49.11 years (±7.78 years); TH-BA—PP: 52.60 years (±9.38 years), P1: 50.76 years (±9.54 years), P2: 51.76 years (±8.91 years); SH—PP: 48.16 years (±9.01 years), P1: 40.33 years (±10.51 years), P2: 43.75 years (±13.12 years); VH—PP: 58.72 years (±10.66 years), P1: 46 years, P2: 51.5 years (±9.32 years); and RH—PP: 57.75 years (±10.75 years), P1: 48 years, P2: 58.66 years (±9.29 years). We observe that the age of addressability to surgical treatment decreased in P1 and tended to increase in P2.

The average age of the patients who underwent M was 37.47 years (±8.01 years). The youngest patients were recorded in P2, 31 years.

The mean age of patients undergoing EL was 32.65 years (±6.34 years), with the youngest age recorded in P1 (26 years); the mean age of LM was 42 years, registered in PP, that of LS was 34.07 years (±6.87 years), with the youngest age recorded in P1 (28 years); that of ULC was 32.2 years (±5.56 years), with the youngest age recorded in P2 (27 years) and that of BLC was 35.36 years (±4.33 years), with the youngest age recorded in P1 (31 years).

The average age of the patients undergoing salpingectomy varied as follows: in TEPS, it was equal to 31.21 years (±5.59 years), with the youngest age recorded in P1 (25 years); in BS, it was 35.76 years (±10.43 years), with the youngest age recorded in P2 (24 years) and in the US it was equal to 36.25 years (±3.30 years), with the youngest age recorded in P1 (32 years).

The mean age of ExP patients was 41.46 years (±12.87 years), with the youngest age recorded in P1 (28 years).

### 3.9. Patient Hemoglobin Levels

**Figure 11:**
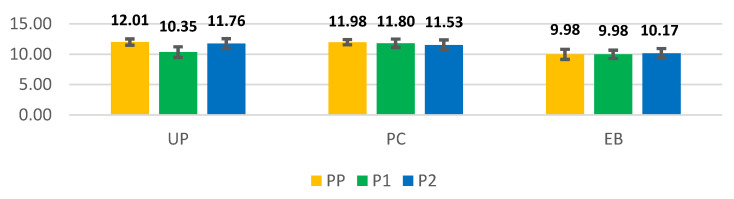
The mean hemoglobin level (g/dL) depends on the pathology category. UP: uterine pathology; PC: pregnancy curettage; EB: hemostatic curettage/endometrial biopsy; PP: the year before the SARS-CoV-2 pandemic (orange columns), P1: the first year of the SARS-CoV-2 pandemic (green columns); P2: second year of the SARS-CoV-2 pandemic (blue columns); US: unilateral salpingectomy; BS: bilateral salpingectomy; TEPS: tubal ectopic pregnancy salpingectomy.

In terms of patient condition at admission, we compared the mean hemoglobin levels (g/dL) of the patients with uterine pathologies (UP), those who underwent PC, and those who underwent hemostatic curettages (EB), depending on the study year. We noticed that in PP, the average values were as follows—UP: 12.01 g/dL (±0.51 g/dL); PC: 11.98 g/dL (±0.43 g/dL); EB: 9.98 g/dL (±0.83 g/dL); in P1, they were as follows–UP: 10.35 g/dL (±0.88 g/dL); PC: 11.80 g/dL (±0.69 g/dL); EB: 9.14 g/dL (±0.68 g/dL); and in P2, they were as follows—UP: 11.76 g/dL (±0.81 g/dL); PC: 11.53 g/dL (±0.83 g/dL); EB: 10.17 g/dL (±0.75 g/dL) (Figure 11).

## 4. Discussion

### 4.1. The Influence of the Pandemic Crisis

The spread of infection with the SARS COV-2 virus started in December 2019 in Wuhan, China, from where it spread to most provinces and later to most countries in the world, resulting in a pandemic [23]. In Romania, the initial infection source was people who came from abroad who had had contact with infected people [24]. Starting on 16 March 2020, Romania declared a state of emergency that lasted until March 2022, when the state of emergency was lifted [25].

The COVID-19 pandemic has left a mark worldwide through the appearance of medical complications, but also through high mortality rates. By 8 December 2022, there had been 6,649,490 deaths worldwide, of which 67,289 were in Romania [26]. This pandemic has also led to restrictive measures and drastic social distancing measures (limiting the number of people in enclosed spaces, limiting travel using public transport, going shopping, limiting travel abroad, and wearing a protective mask) to reduce the viral spread. All these measures had consequences on family dynamics, income, interpersonal relationships, and on mental well-being and health [27].

Women’s health has been affected during the pandemic due to a decrease in or lack of access to doctors, fiscal deficit, and by restrictions in the medical system, while effects due to hormonal changes caused by stress, psychological suffering, dietary patterns, changes in physical activity, and long-term implications will continue to be seen in the coming years [27,28]. More studies are necessary to assess the effect over time and to truly understand the consequences of the lack of access to physicians during the pandemic [28].

The vaccination campaign started in Romania in December 2020, which led to a gradual decrease in the number of confirmed cases of illness. We expected that population vaccination would be accompanied by a marked increase in addressability for gynecologic conditions, following the restrictions of the first pandemic year, where there was an obvious decrease in the number of interventions. In December 2021, our country was the only country in the European Union in the green zone in the list of countries with high epidemiological risk. The cumulative incidence rate per 1000 inhabitants in Romania was 1.1 [29].

### 4.2. The Impact of the Pandemic on Gynecological Interventions 

#### 4.2.1. The Impact of the Pandemic on Interventions for Ovarian Pathology

In this study, we aimed to assess the impact of the pandemic on the care of patients with various pathologies requiring surgery, comparing PP with P1 and P2 (including PV). In our study, we observed that the number of adnexectomies decreased by half in P1 and by one-third in P2 compared to PP. Other studies have shown that the SARS-CoV-2 virus has had and will have a potentially damaging effect on the ovarian structure because COVID-19 disease is an independent risk factor in terms of ovarian function as a result of hormonal changes (levels of the anti-Mullerian hormone, testosterone, and prolactin) [30,31], the “immune/inflammatory distortion” caused by SARS-CoV-2, and because of the lack of access to doctors for surgical interventions during the pandemic of women diagnosed with ovarian tumor formations [31].

The slight increase in the number of UA-type interventions in P2 can be explained by the decrease in the number of diseases in PV, but also by the anxiety, depression, or insomnia present during the pandemic, which can cause an increase in prolactin levels and implicitly cause dysfunction of the hypothalamic–pituitary–ovarian axis. In terms of increased secretion of the luteotropic hormone, it has been proven that it stimulates the cells to synthesize more testosterone and aggravates polycystic ovary syndrome or causes the appearance of large ovarian cysts that require surgery. Additionally, the number of interventions in PV did not return to the PP level, because patients were still afraid of the public healthcare system, and they more frequently chose private practices and more conservative protocols [32].

#### 4.2.2. The Impact of the Pandemic on Pregnancy Curettage or Incomplete Abortion

In our study, we observed that during P1 and P2, the number of pregnancy curettage procedures decreased by about one-third compared to PP. This decrease can be explained by patients’ fears of going to the hospital during their miscarriage. Studies have shown that pregnancy is an aggravating factor for COVID-19 disease [33,34,35,36,37], but pregnant women also have an increased risk of abortion and premature birth [37,38,39,40,41]. The abortion rate increased in combination with the SARS-CoV-2 virus [42,43,44], and the potential for vertical transmission from mother to embryo/fetus has been demonstrated since the beginning of P1 [14,44,45,46]. If infection occurs in the early stages of the pregnancy, especially during organogenesis, the miscarriage rate increases [47,48] due to the inflammation and placental insufficiency caused by the virus’s effect on the placental structure [34,37]. Recent research has shown the lodging of the intervillous space with fibrin and the appearance of multiple villi infarcts in mothers infected with SARS-CoV-2; this affects the transport of nutrients from mother to fetus and may be associated with adverse effects on pregnancy [49].

However, we noted a decreased number of miscarriages and pregnancy curettages in our public unit, which may be due to the restricted interaction between the individuals, decreased social relations, and the fear of pregnant women being admitted to public hospitals for the necessary care. Moreover, the limited collection of fetal and placental samples for research, due to the lack of the mothers’ consent, the lack of SARS-CoV-2 diagnostic tests, and epidemiologic restrictions, hampered histopathological investigations and limited the current data on the reported cases [50].

#### 4.2.3. The Impact of the Pandemic on Hemostatic Curettage or Endometrial Biopsy, Biopsies Performed for Various Vaginal or Vulvar Lesions and Polypectomies

With regard to gynecological care, a decrease in outpatient addressability was observed during the COVID-19 pandemic, and the prevention of malignant transformation of various gynecological tumors and early diagnosis are of paramount importance in our specialization. Still, the European Federation for Colposcopy (EFC) and the European Society of Gynecological Oncology (ESGO) have recommended that vaccination programs, screening programs, colposcopies, and human papillomavirus (HPV) detection be rescheduled to a safer time of the global health crisis, in an attempt not to compromise the safety of patients effectively [51].

However, according to EFC and ESGO, since the imposition of restrictions regarding diagnostic procedures for pre-invasive and elective therapeutic lesions by genital biopsies (vaginal, vulvar, endometrial), the rate of abnormal results has decreased, and implicitly, the risk of lesion progression has increased [52].

Our study showed a marked reduction in the total number of biopsies, by almost half, in P1 compared to PP, especially in April and November, and by one-third in P2 compared to PP. Studies have shown that hyperestrogenism can cause hyperplastic endometrial transformation, with the development of polyps or the thickness of the endometrium, a condition that can appear, for example, in polycystic ovary syndrome. In the section above, we reported that psychological factors, stress, and living conditions during the pandemic disrupted the hypothalamic–pituitary–ovarian axis and increased the risk of aggravation of polycystic ovary syndromes and, implicitly, the occurrence of hyperestrogenism [30,31], thus explaining a possible cause of the increase in the percentage of biopsies/polypectomies performed in P2 compared with P1. Another potential explanation would be the doctor’s or patient’s preference for conservative interventions in endometrial conditions, instead of radical approaches such as hysterectomy, during the first pandemic year.

Regarding EB, VB, VuB, we noticed a decrease in P1 and P2 compared to PP. The percentage of EB decreased by more than half in P1 and by about a third in P2 compared to PP; the percentage of VB was the same in P1 and PP, but decreased by almost half in P2 compared to PP and P1, while the percentage of VuB also decreased by half in P1 and P2 compared to PP. The percentage of polypectomies decreased by two-thirds in P1 and about three-quarters in P2. All of these changes were probably due to the temporary restrictions regarding procedures not constituting a surgical emergency. In endometrial high-grade lesions associated with vaginal bleeding that do not respond to hemostatic treatments, it is recommended to postpone the elective intervention by a maximum of 2–4 weeks, in the case of contraindications to the administration of hormonal therapy and for 10–12 weeks in the case of minor injuries [52]. In the case of suspicious vulvar or vaginal lesions, the excisional biopsy can be postponed for a maximum of 2–4 weeks in the case of high-grade lesions and for 10–12 weeks in cases of vulvar and vaginal intraepithelial neoplasia cases [52].

#### 4.2.4. The Impact of the Pandemic on Interventions Performed for Various Uterine Pathologies (Leiomyoma/Adenomyosis/Cancer/Ovarian Pathologies in Perimenopause)

In the course of this study, we noticed a steep decrease in the number of gynecological surgeries such as hysterectomies (TH-UA, TH-BA, SH, VH, RH) or M, performed during the pandemic. The reduction in surgical volume was more evident during P1, when the most restrictive institutional and regional policies were applied, prohibiting or drastically restricting elective surgical interventions during the pandemic.

However, a slight increase in the number of surgical interventions such as hysterectomies was observed in P2 (PV), although it did not reach PP levels. Additionally, ambulatory care decreased substantially in P1, and failed to once more reach the PP level, which has also resulted in a decrease in revenues allocated to hospitals for gynecological care. The financial impact of such losses has been substantial, as many health systems operate with low operating margins [53]. Surgical gynecological practices during P1 and P2 were likely influenced by published guidelines regarding surgical practice during the COVID-19 pandemic [54,55,56]. Additionally, there may be a migration of the population towards alternative private health care, which the population may have accessed for a potentially greater degree of medical safety during the COVID pandemic.

In our center, uterine pathologies (adenomyosis, leiomyoma) associated with persistent metrorrhagia and menometrorrhagia were initially medically managed, and their treatment was postponed or they were operated on depending on the severity of the condition. In the case of confirmed malignant pathologies, depending on staging, an attempt was made to offer rapid surgical treatment for predisposed patients and subsequent oncological treatment. The French Society of Obstetrics and Gynecology (CNGOF) issued, through the FRANCOGYN group on 23 March 2020, a guide for oncological treatment during this state of emergency. In the case of cervical malignancy, this guideline oscillated between radio-chemotherapy and surgical intervention, with the reserved practice of lumbar-aortic lymphadenectomy due to the associated immunosuppression risks [57,58]. In the case of endometrial cancers, CNGOF recommended postponing interventions for those at low risk or without related comorbidities, while for those at high risk, imaging exploration and surgical intervention were recommended. In the case of ovarian cancers, neoadjuvant chemotherapy was most often practiced [57]. Comparatively, our center surgically treated (RH with lymphadenectomy) predisposed oncological patients after performing an imaging exploration in all cases registered during the P1 and P2 periods.

Currently, there are few data available regarding the effect of the pandemic on cancer patients and their survival rate. These will be retrospectively evaluated in future studies. Our study showed an estimated decrease in the number of oncological interventions performed in our center, by over 90% in P1 and by more than 80% in P2 compared to PP.

In the study by Martinelli F. et al., information was gathered from 49 countries regarding the management of gynecological cancers, and it was shown that 30–40% of cases were treated with chemotherapy, while the number treated using laparoscopic surgery decreased by 30%, and radiotherapy treatment increased by 24%. In the case of low-risk endometrial cancers, surgical intervention was postponed, and neoadjuvant hormonal therapy was used. In the case of ovarian cancer, most of the interventions were postponed, and neoadjuvant chemotherapy was also used. In the case of early stage cervical cancers, the intervention was postponed in 15% of cases. All of these figures, as well as the decrease in the number of cases in P1 and P2, confirm the considerable impact of the COVID-19 pandemic on the care strategy for oncology patients [59].

#### 4.2.5. The Impact of the Pandemic on Laparoscopic Interventions

While we noted that elective interventions were limited or postponed during the COVID-19 pandemic, gynecological emergencies still had to be managed. The type of intervention technique employed (classical or laparoscopic) must that which offers the greatest possible degree of surgical safety and the lowest associated risk of viral spread [60]. During the pandemic period, it was necessary to evaluate gynecological surgical emergencies correctly and the type of intervention performed, laparoscopic or by laparotomy. In our center, most laparoscopic interventions were performed during PP; their number was reduced by 3.6 times in P1 and 1.71 times in P2.

The American Association of Gynecologic Laparoscopists (AAGL), along with other societies such as the American College of Obstetricians and Gynecologists (ACOG), recommended the postponement of elective interventions in the first part of the pandemic [61], which explains the decrease in the total number of interventions performed at our center. Concomitantly, the Royal College of Obstetricians and Gynecologists (RCOG) and the British Society for Gynecological Endoscopists (BSGE) promoted laparoscopic interventions based on their benefits in terms of recovery, as well as on better use of hospital waste during the time of the pandemic [62]. The European Society for Gynecological Endoscopy (ESGE) supported the postponement of elective interventions for benign pathologies with the promotion of drug treatment, and exploratory laparoscopy as the appropriate approach in cases of gynecological emergencies [63]. Still, there are no data regarding variations in the number of gynecologic laparoscopic procedures in Europe during the COVID pandemic.

#### 4.2.6. The Impact of the Pandemic on Exploratory Laparotomies

Cohen SL et al. argued that during the pandemic, patients who required urgent surgical intervention and did not have enough time for preparation should be given an exploratory laparotomy to minimize the risks of delaying the operative act. The availability of surgical approaches is conditional on in-hospital equipment, the training of the medical staff, and the condition of the patients [64]. In this study, a small decrease in the number of laparotomies in P1 (by 11.1%) was observed, and an increase of more than half in P2 (63.28%) compared to PP. One explanation for these figures could be the much more careful investigation of gynecological conditions proposed for safety reasons to establish diagnosis before operative intervention.

#### 4.2.7. The Impact of the Pandemic on Interventions Performed for Tubal Pathology (Tubal Ectopic Pregnancy or Pelvic Inflammatory Disease)

A total of 85% of cases of ectopic tubal pregnancy (TEP) can be diagnosed before they become complicated with hemoperitoneum and require emergency surgery [65]. The traditional treatment is surgical, because TEP can endanger maternal life, but nowadays, early diagnosis using serum human chorionic gonadotropin (β-hCG) dosage and high-resolution transvaginal ultrasound considerably reduced maternal mortality. Thus, radical surgical interventions for TEPs such as salpingectomy have been significantly reduced, and the rate of medical treatment with methotrexate or expected management has increased [66,67,68]. In our study, the number of TEPs decreased by three-quarters in P1 and by more than half in P2 compared to PP, suggesting that the number of tubal ectopic pregnancies decreased or that more patients adhered to drug therapy or private practice. These recommendations probably decreased the percentage of surgical interventions for ectopic tubal pregnancy. We believe that reduced availability of medical services and women’s fear of being exposed to hospital-acquired infection reduced medical care and checkups. The results of our study are consistent with other reports showing reductions in emergency medical services in P1 and P2 during the COVID-19 pandemic [69,70,71].

The number of BS increased by 50% during P1 and P2, and the number of US decreased by more than a third in P1 and halved in P2 compared with PP, probably due to the difficulties in following up young girls diagnosed with pelvic inflammatory disease [72]. Issues of confidentiality, lower education, and lack of interest when it comes to young patients with regard to sexual education and condom use represent primary intervention targets to minimize complications from sexually transmitted diseases, especially during the COVID-19 pandemic. Currently, this information is available to most young people, and a strategy for preventing the spread of sexually transmitted infections causing PID is feasible [73].

### 4.3. The Impact of the Pandemic on the Age of Patients

During the pandemic, the average age of patients varied naturally from 30.69 years (±8.22 years) for PC to 69.4 years (±14.91 years) for VuB-type interventions. Neoplastic pathologies were admitted, as expected, at advanced ages. The age of patients with PC, BA/AU decreased in P1 and P2 compared to PP. The average age of biopsied patients also decreased in P1 and P2 compared to PP. The age of the patients that underwent a hysterectomy was lower in P1 and increased slightly in P2. In the case of M and EL, the average age of patients decreased during P2, and decreased in TEPs mainly during P1.

Thus, in almost all types of intervention, the average age of patients decreased during the COVID-19 pandemic, demonstrating that young patients presented to the doctor more frequently relative to elderly women, probably as a result of media reports. Bearing in mind the general decrease in the number of gynecologic surgical interventions administered, it is obvious that older patients were more influenced by media information regarding the age-related severity of SARS-CoV-2 infection, leading to a decrease in their medical addressability [73].

### 4.4. The Impact of the Pandemic on the Hemoglobin Levels of Patients

We observed in our study that during the PP period, in UP cases (leiomyomas, adenomyosis, cancers), the average hemoglobin level was 12.01 g/dL, compared to P1 and P2, when the average levels decreased to 10.35 g/dL and 11.76 g/dL, respectively. These findings suggest that the patients delayed seeking medical care in cases of vaginal bleeding, particularly in the first part of the pandemic, probably due to the media reports informing the population about the viral spread and COVID consequences [73]. We did not observe significant oscillations in the average hemoglobin levels of the patients presenting for miscarriage requiring evacuation curettage. In these cases, the average hemoglobin levels varied from 11.98 g/dL in PP to 11.8 g/dL in P1 and 11.53 g/dL in P2. Similarly, in the case of EB, the average hemoglobin levels exhibited comparable levels: 9.98 g/dL in PP and P1, and 10.17 g/dL during P2 [74].

### 4.5. Limitations and Considerations Regarding this Study

This study presents several limitations that need to be taken into account. Firstly, the limited length of the study, 3 years, means that conclusive results on all aspects of the influence of the SARS-CoV-2 pandemic on the number of chronic gynecological interventions in Romania cannot be provided, especially considering the fact that the pandemic was still ongoing even following the end of our statistical study. Another limitation was linked to the methodology of the study, in that not all of the identified cases of various pathologies were treated surgically in public hospitals. This was a result of the increasing access made by patients of private medical facilities in order to avoid public facilities. Similar to other studies [4], another limitation of this research was the retrospective design (reported cases), and reference to future studies will be required in order to obtain comparative results. Another limitation of the study is that we do not know how many patients were infected before their procedures or how many patients were vaccinated before or after their procedures; therefore, we do not know what side effects could have impacted the outcomes of gynecological pathologies.

Overall, we observed a marked decrease in gynecological interventions in a county hospital in Romania during the SARS-CoV-2 pandemic period compared to the pre-pandemic period. This decrease in the number of surgical interventions was more accentuated during the first part of the pandemic, when severe hospital restrictions were imposed, and elective interventions were delayed. Subsequently, after the onset of vaccination and the easing of restrictions, we observed that the number of surgeries started to increase, but not to pre-pandemic levels, as shown in previous discussions, and as shown by other similar studies reporting substantial financial losses [75].

Surgical gynecological services in our center during this public health crisis were substantially influenced by guidelines published by the medical society regarding changes in surgical practice during the SARS-CoV-2 pandemic [76,77,78].

The data in our study did not allow us to analyze and address the impact of postponing or delaying medical care, but we were able to observe some percentage decreases in the number of biopsies and other interventions, similar to what has been reported in other studies, where decreases in numbers of mammograms [79,80], screening tests for sexually transmitted infections [81], and Babes Papanicolaou smear screening tests were reported [82].

The medical community has been concerned about the negative health outcomes of delayed medical care and associations have been made with the initial diagnosis of breast or cervical cancers at more advanced stages [83]. Reduced access to diagnostic tests for genital infections will lead to a return of their incidence [84]. The decrease in the proportion of gynecological interventions raises questions about the impact on long-term health, but mitigation of these effects would be possible through increased use of telemedicine. It has been demonstrated that telemedicine impacted gynecological and obstetric outcomes regarding breastfeeding, access to medical abortion, use of oral contraception and the need for high-risk obstetric visits [85].

## 5. Conclusions

The COVID-19 pandemic played a significant role in decreasing elective surgeries, especially before the vaccination. Due to the healthcare system restrictions, the number of patients undertreated is significant, and the actual effect of the pandemic is still to be seen. During the SARS-CoV-2 pandemic, the patients apparently avoided public hospitals in Romania due to the healthcare system restrictions, and fear of contracting COVID, leading to a low rate of elective gynecologic surgeries during the pandemic years. The average age of those patients surgically treated decreased during the first two pandemic years because of the lower addressability of the elderly women under the influence of the media reports. The average hemoglobin levels at admission decreased during the pandemic, and especially in the first year, as the patients delayed accessing medical care in cases of vaginal bleeding.

## Data Availability

Data was collected from the Emergency University County Hospital of Craiova, from the Obstetrics–Gynecology Clinics. These data are not available online, but can be provided upon formal request.
